# Transcranial ultrasound stimulation in neuromodulation: a bibliometric analysis from 2004 to 2024

**DOI:** 10.3389/fnins.2025.1595061

**Published:** 2025-05-13

**Authors:** Jingxuan Wang, Yuling Wang, Shuyan Qie

**Affiliations:** ^1^Beijing Rehabilitation Medicine, Beijing Rehabilitation Hospital, Capital Medical University, Beijing, China; ^2^Department of Orthopedics II, Beijing Rehabilitation Hospital, Capital Medical University, Beijing, China; ^3^Department of Rehabilitation, Beijing Rehabilitation Hospital, Capital Medical University, Beijing, China

**Keywords:** physiotherapy, transcranial ultrasound stimulation, non-invasive brain stimulation, neuromodulation, bibliometric analysis

## Abstract

**Background:**

Transcranial ultrasound stimulation (TUS) is a non-invasive neuromodulation technique with promising clinical potential. Its therapeutic efficacy and safety are significantly influenced by stimulation parameters. However, the global research hotspots and future research trends of TUS application in the field of rehabilitation are unclear. This study analyzes the status of TUS research. Understand the annual publication trends, international and institutional cooperation pattern and influential authors and journals and keyword hotspot.

**Methods:**

A comprehensive literature search was conducted on the Web of Science core database using TUS-related subject headings until 27 December 2024. Two researchers independently screened articles based on pre-determined inclusion and exclusion criteria. Software packages such as CiteSpace and VOSviewer were used to visualize the results.

**Results:**

A total of 577 literatures were included. The results show that the annual publication volume shows an increasing trend, reaching a peak in 2024. The United States, China and Germany dominated the number of publications, with the largest number of institutions being Harvard University, the University of Toronto and Brigham and Women’s Hospital. Brain stimulation is the journal with the most articles and citations. Research hotspots include transcranial magnetic stimulation, noninvasive brain stimulation, Parkinson’s disease, and Alzheimer’s disease.

**Conclusion:**

A bibliometric analysis of the literature shows that research interest in transcranial ultrasound stimulation is growing rapidly, with annual publications growing exponentially since 2013 and receiving increasing attention from researchers. The findings suggest that TUS is currently used primarily in neurological diseases, particularly in the study of Parkinson’s disease and Alzheimer’s disease. At the same time, it is found that an emerging international cooperation model with the partnership between the United States, China and Germany as the core has gradually taken shape. Although preclinical studies have shown promising neuromodulator effects, the current study suggests that TUS needs to undergo further multicenter clinical validation. These findings provide evidence to guide future research priorities for non-invasive neuromodulation.

## 1 Introduction

Non-invasive brain stimulation (NIBS) is a widely used neuromodulation technique that employs physical stimuli to regulate neural activity, offering a safe and minimally invasive approach with few side effects (Penton et al., 2022). Transcranial Ultrasound Stimulation (TUS) is an emerging neuromodulation technology that utilizes ultrasound waves to modulate neuronal activity (Darmani et al., 2022).

TUS operates through the mechanical and cavitation effects of ultrasound (Plaksin et al., 2016), which induce deformation of neuronal cell membranes (Prieto et al., 2018), change in ion channel permeability (Sorum et al., 2021; Oh et al., 2019; Yoo et al., 2022), and modulate neuronal excitability (Blackmore et al., 2021). Unlike invasive techniques, TUS achieves high spatial resolution and can target deep brain regions with minimal off-target effects (Blackmore et al., 2019). The effective depth of TUS can reach 5 ~ 7 cm below the cortical surface, enabling targeted stimulation of subcortical structures that are otherwise inaccessible to conventional techniques (di Biase et al., 2019). Currently, TUS is adaptable to various clinical and research applications, including neurodegenerative diseases (Nicodemus et al., 2019; Jeong et al., 2021; Jeong et al., 2022; Shimokawa et al., 2022; Popescu et al., 2021; Beisteiner et al., 2020; Cont et al., 2022; Dörl et al., 2022; Matt et al., 2022; Wojtecki et al., 2024), psychiatric disorders (Reznik et al., 2020; Cheung et al., 2023a; Oh et al., 2024; Schachtner et al., 2024), and cognitive enhancement (Fong et al., 2023; Cain et al., 2022). As research progresses, TUS has the potential to treat neurological and psychiatric disorders, and offering new hope for patients.

Tyler et al. (2008) found that low-intensity transcranial ultrasound stimulation (LITUS) had an excitatory regulation effect on neuronal activity and brain network, which laid an important foundation for subsequent related research. Hameroff et al. (2013) first applied non-focused ultrasound to the posterior frontal cortex of patients with chronic pain. The results showed that compared with the placebo group, treated patients in the two aspects of pain and emotional have improved significantly. This exploratory study initially demonstrates the great potential of TUS as a clinical treatment method for NIBS. TUS as an emerging non-invasive neuroregulatory technology, its clinical applications and research methods have only developed rapidly in the past two decade, although a large number of independent studies have explored its mechanism or efficacy, but the lack of quantitative integration of the overall research landscape, resulting in hot spots and collaborative networks have not been systematically revealed (Darmani et al., 2022).

Bibliometric analysis is the quantitative analysis and processing of bibliographic data using mathematical and statistical methods to provide a systematic framework for understanding the current state of research, mapping scientific progress, and predicting future directions (Hicks et al., 2015). Previous studies have investigated the mechanisms and clinical efficacy of TUS (Sarica et al., 2022; Qin et al., 2024; Lee et al., 2024), but few studies have integrated global output in the field, interdisciplinary collaborations, or temporal changes in research priorities.

This study conducted a comprehensive bibliometrics analysis of the articles included in the Web of Science Core Collection database from 2004 to 2024. Using CiteSpace (Chen, 2006) and VOSviewer (van Eck and Waltman, 2010) visualization tools to understand the annual publication trends, international and institutional cooperation pattern and influential authors and journals and keyword hotspot, help the researchers to understand the research hotspot in the field of TUS and predict the future trend (Glänzel and Czerwon, 1995).

## 2 Materials and methods

### 2.1 Data sources

The data for this study were sourced from the Web of Science Core Collection (WoSCC), one of the most utilized databases for bibliometric analysis. The search period spanned from 2004 to December 27, 2024. A 20-year span ensures robust trend analysis while minimizing database indexing gaps common for early-stage. The detailed WoSCC search strategy is presented in Table 1. A total of 591 literatures related to TUS were retrieved using these keywords.

**Table 1:** Search strategy in WoSCC.

Step	Retrievable
#1	(ALL = (non-invasive brain stimulation)) OR ALL = (noninvasive brain stimulation)
#2	(ALL = (transcranial ultrasound stimulation)) OR ALL = (transcranial ultrasound)
#3	ALL = (TUS)
#4	(ALL = (focused ultrasound stimulation)) OR ALL = (focused ultrasound)
#5	(ALL = (transcranial focused ultrasound stimulation)) OR ALL = (transcranial focused ultrasound)
#6	(ALL = (transcranial unfocused ultrasound stimulation)) OR ALL = (transcranial unfocused ultrasound)
#7	ALL = (transcranial pulse stimulation)
#8	#2 OR #3 OR #4 OR #5 OR #6 OR #7
#9	#1 AND #8

### 2.2 Inclusion criteria and exclusion criteria

Inclusion Criteria: ① Studies focusing on the application of TUS in the field of rehabilitation; ② Literature types limited to reviews or clinical studies. Exclusion Criteria: ① Non-research publication types: Editorials (opinion pieces without data analysis); Letters (short communications not presenting original research); Meeting abstracts (conference materials without full methods/results); News reports, books, and patents. ② Duplicate publications or incomplete data. ③ Studies with incomplete information or those for which full text could not be obtained.

### 2.3 Study extraction

Study extraction was independently performed by two researchers. Initially, preliminary screening was conducted based on the titles and abstracts of the articles, followed by a secondary screening according to the inclusion and exclusion criteria. In cases of disagreement, a third researcher was consulted to reach a consensus. The main characteristics of each included study were extracted, including the title, authors, institutions, and keywords.

### 2.4 Data extraction and data preprocessing

The retrieved data were exported in RefWorks format. Data cleaning was first performed to remove erroneous, incomplete, or irrelevant data, including the identification and correction of spelling errors in author names and publication years. Subsequently, institutional names were checked and standardized, with different representations of the same institution unified to the most recent and standardized form, such as “Peking University” standardized from “PKU” or “Beijing University.” For keyword processing, similar expressions were consolidated to ensure consistency, and ambiguous terms were manually reviewed for accuracy, such as “TUS” and “Transcranial ultrasound stimulation.”

The data were saved in the format of download.txt. The data were imported into CiteSpace v6.2.R6, where duplicate articles were excluded based on title and DOI. For tools requiring specific input formats, data were reorganized to match field requirements. Missing values in critical were marked and excluded from relevant analyses to avoid bias. The reasons for exclusion included editorial materials, letters, meeting abstracts and so on.

### 2.5 Data analysis and visualization

The included literature was subjected to visual analysis using CiteSpace 6.2.R4, VOSviewer 1.6.20, Scimago graphica, Charticulator and Microsoft Excel. Encompassing annual publication volume, institutional analysis, keyword co-occurrence, and cluster analysis. Nodes were selected based on the type of analysis to be performed (Xu et al., 2021), with the following parameter settings.

For the CiteSpace 6.2.R4: ① Time span: January 2004 to December 2024, with a time slice of 1 year; ② Term sources: Title, Abstract, Author Keywords, and Keywords Plus; ③ Node types: Author, Institution, Country, Keyword, Reference, Cited Author, and Cited Journal. ④ Threshold settings: Minimum co-occurrence count of 5 for keywords and pruning of merged networks to enhance visual clarity.

For the VOSviewer 1.6.20: ① Similarity matrix calculated using the cosine metric for keyword co-occurrence; ② Cluster resolution set to 1.0 to align with CiteSpace’s modularity Q values for cross-tool consistency. For the Microsoft Excel: ① Data normalization for statistical charts; ② Conditional formatting applied to highlight trends in annual publication volumes.

## 3 Results

### 3.1 Selection process and reasons for exclusion

**Figure 1:**
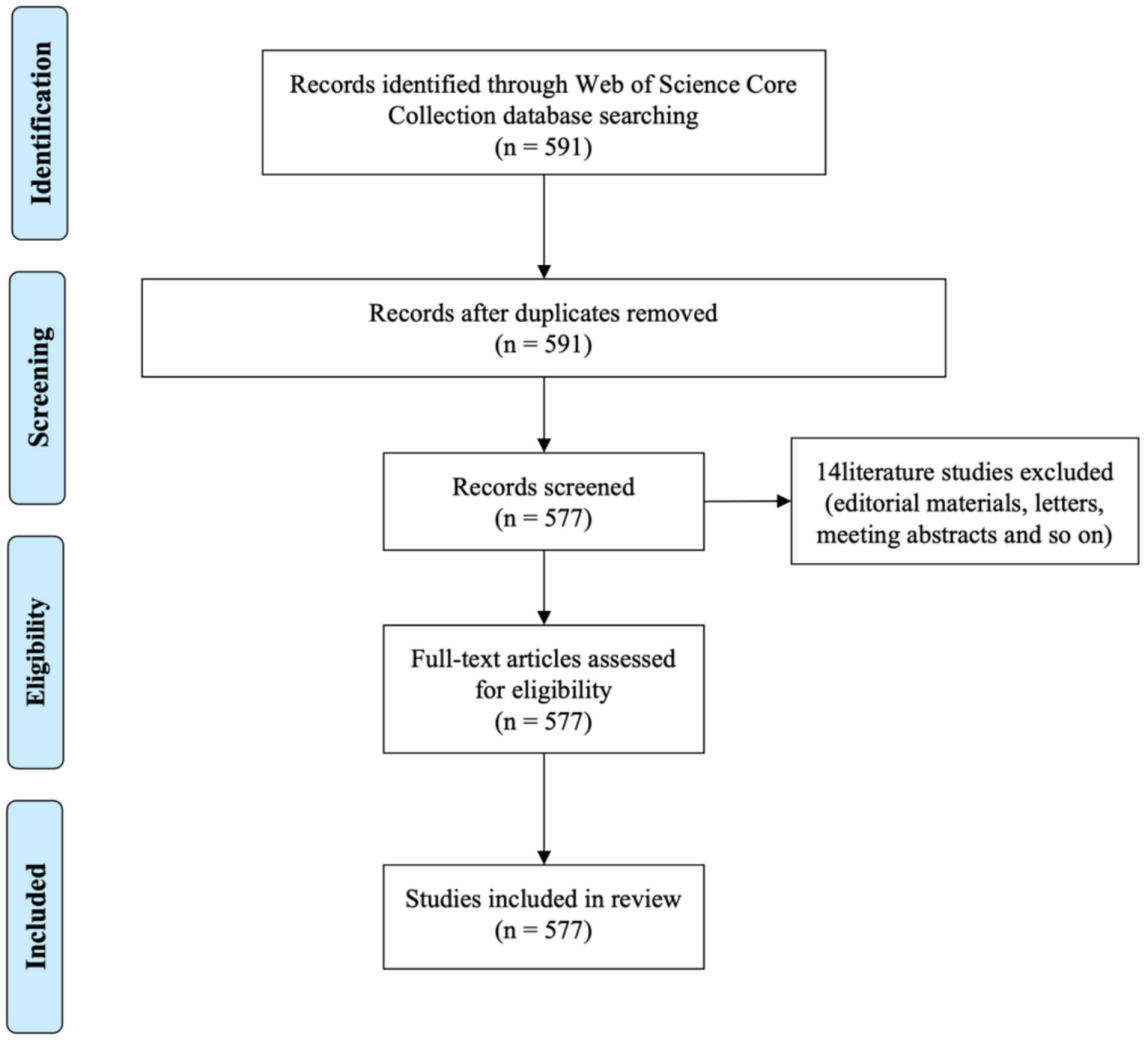
Selection process and reasons for exclusion.

A total of 577 papers were ultimately included in the analysis. The screening process is illustrated in Figure 1.

### 3.2 Distribution of annual publication volume

**Figure 2:**
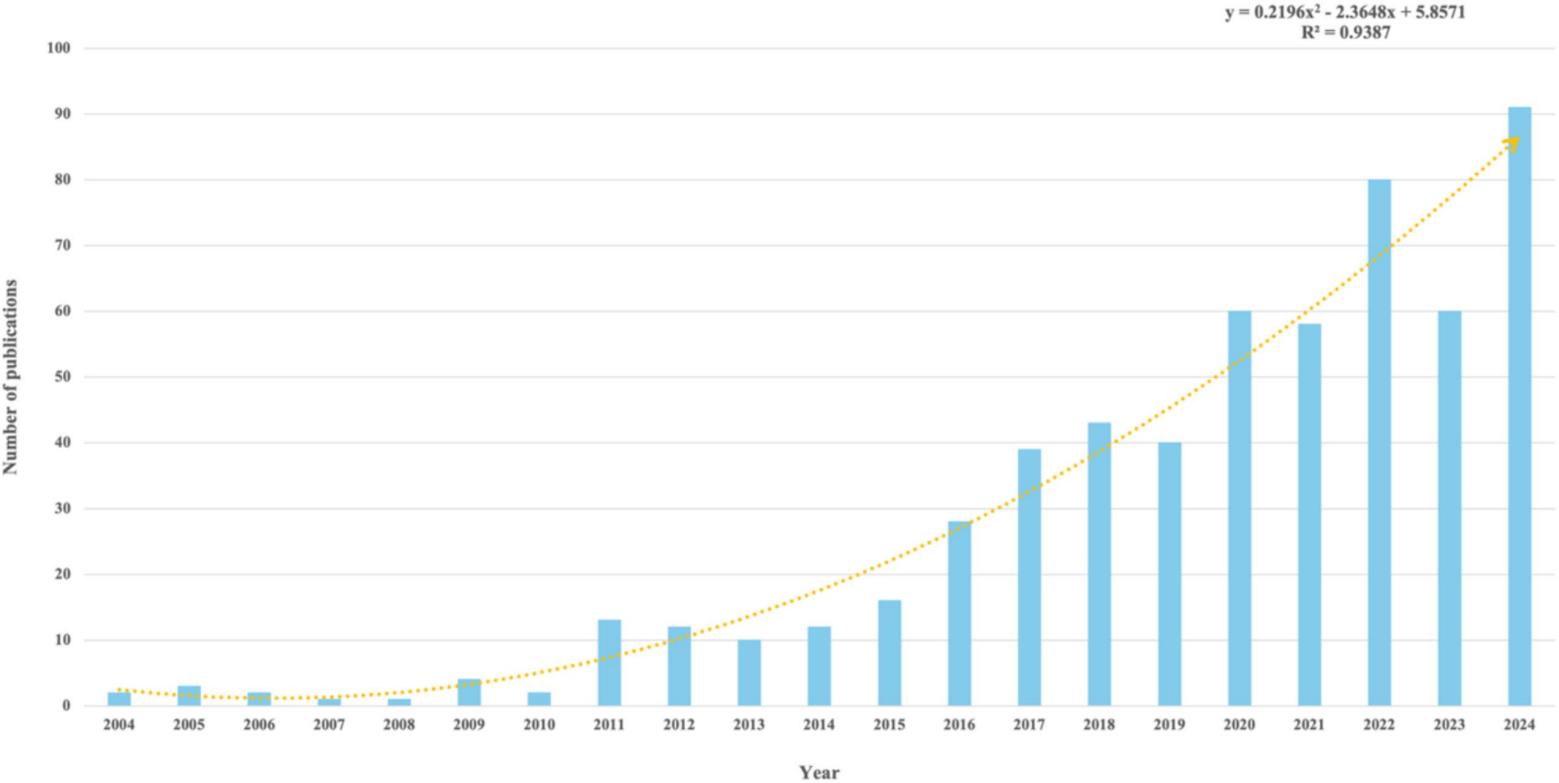
Annual publication volume from 2004 to 2024.

The distribution of annual publication quantity serves as a critical indicator of evolving trends in the field. Since 2004, the number of publications focusing on TUS has demonstrated an overall upward trend. In the early years, publication counts were relatively low, and the growth trend remained relatively indistinct. However, a marked increase became evident over time. Between 2013 and 2024, research output exhibited a sustained upward trend, with minor declines observed in 2019, 2021, and 2023. The publication volume peaked in 2024 at 91 articles, reflecting growing scholarly interest in TUS. The detailed information is showed in Figure 2.

To quantitatively characterize this trend, the annual publication data were fitted with a quadratic growth model using Excel. The derived equation is:


$$y=0.2196x^{2}–2.3648x+5.8571\;(R^{2}=0.9387)$$

where *y* represents the publication count and *x* denotes the year. The positive coefficient of the quadratic term (0.2196) indicates a parabolic upward trajectory, suggesting that future publication volumes are projected to follow an accelerated growth pattern. This robust model fit (*R*^2^ > 0.9) further validates the reliability of the observed trend.

### 3.3 Countries and regions cooperation network map

**Figure 3:**
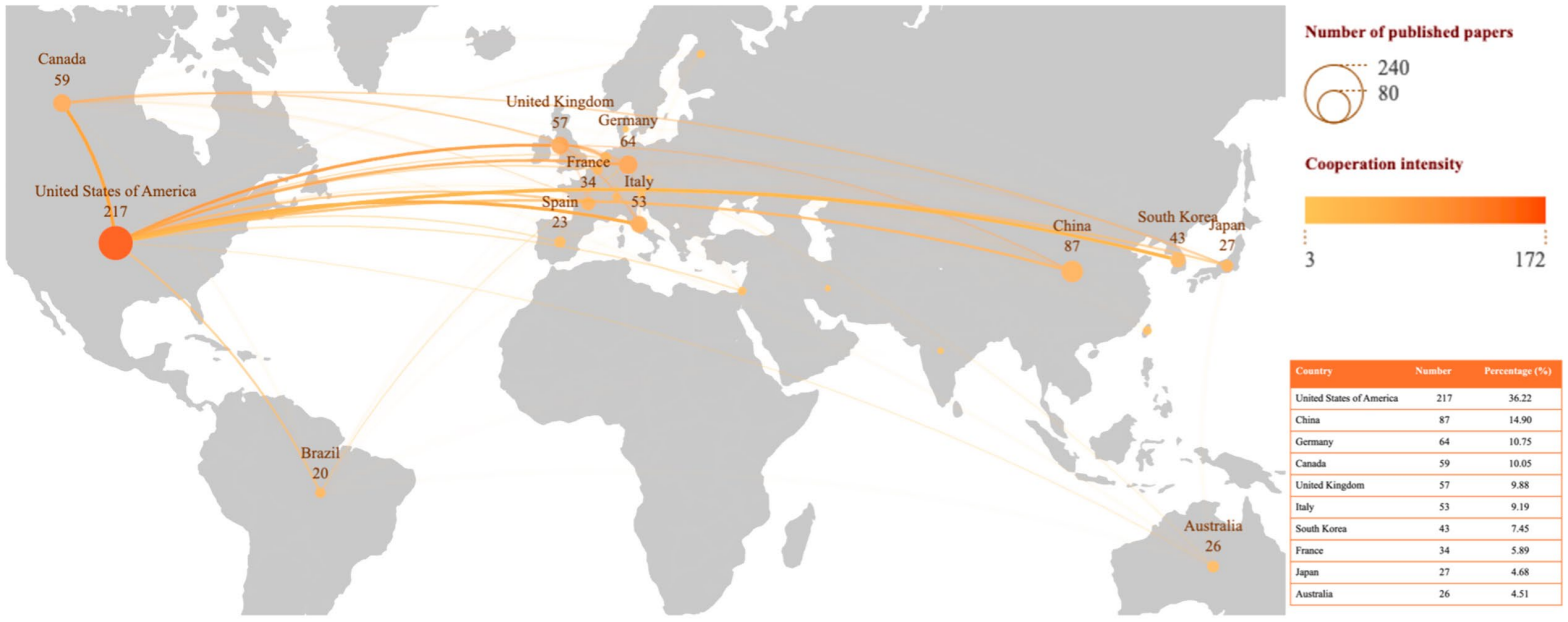
The top 10 productive countries of the topic.

**Figure 4:**
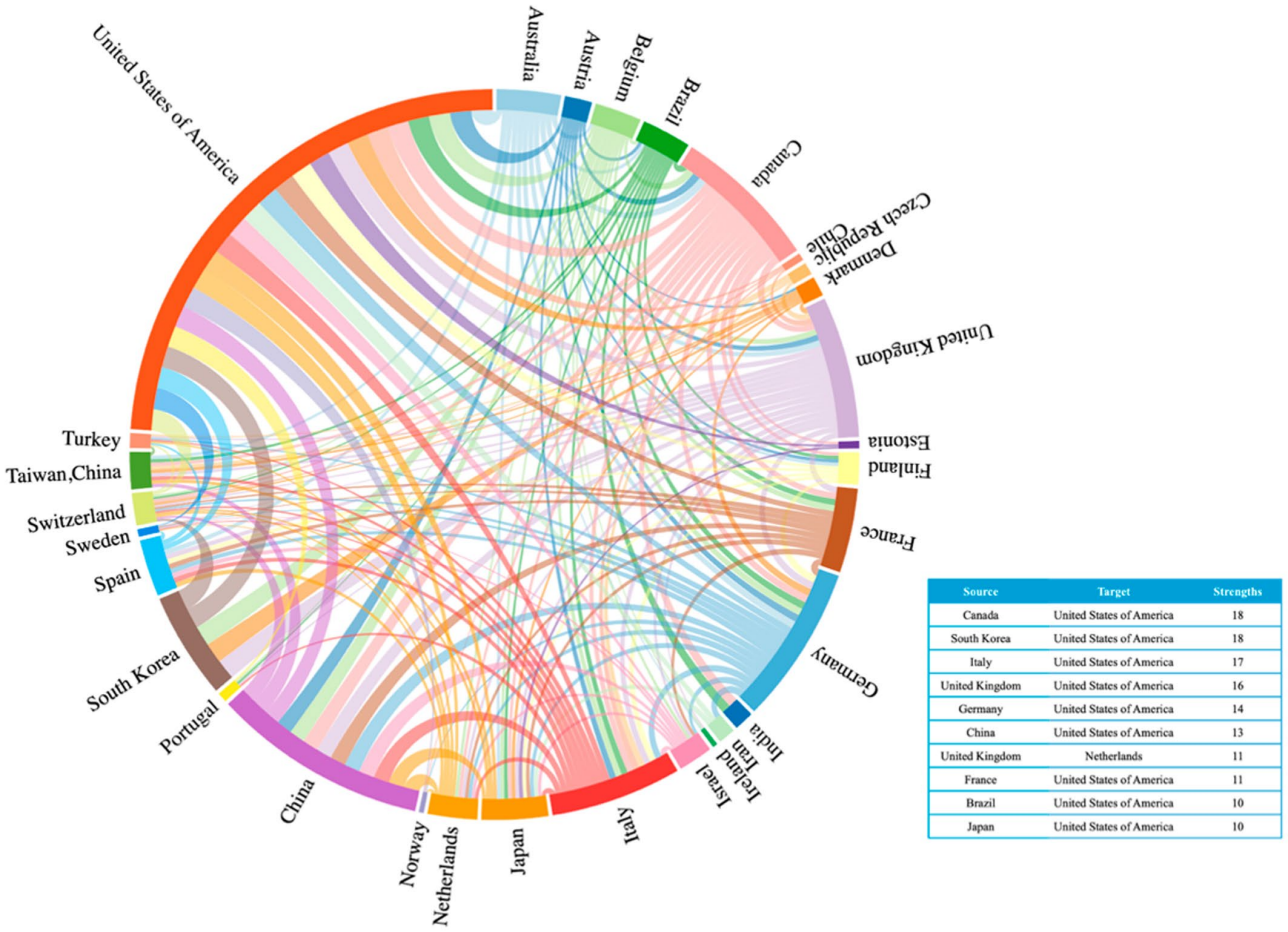
Top 30 countries chord diagram of collaboration strength.

A total of 54 countries have contributed to the publication of articles on TUS. As shown in Figure 3, the United States leads in the number of publications in this field (*n* = 217; 36.22%), followed by China (*n* = 87; 14.90%) and Germany (*n* = 64; 10.75%). This distribution highlights the significant contributions of these countries to TUS research and underscores the global collaborative nature of scientific advancements in this area. Figure 4 illustrates the collaborative network among countries, highlighting the top 10 international/interregional collaboration strengths. The visualization demonstrates the extent and intensity of research partnerships, with prominent collaborative hubs centered around leading countries such as the United States, Canada, and South Korea. This network analysis underscores the global interconnectedness of research efforts and identifies key players driving international collaboration in the field.

### 3.4 Institutions cooperation network map

Table 2 lists the top 10 institutions based on their publication output and centrality within the collaboration network. In terms of publication count, the top three institutions are Harvard University, University of Toronto, and Brigham & Women’s Hospital. Node centrality, which measures an institution’s influence and connectivity within the network, ranks Harvard University, University of London, and Krembil Research Institute as the top three. This disparity highlights Harvard University’s dual dominance in both productivity and collaborative reach, while institutions demonstrate stronger integrative roles in fostering interdisciplinary partnerships. High centrality values suggest these institutions act as pivotal hubs for knowledge exchange and collaboration in TUS research.

**Table 2:** The top 10 institutions according to publications of the topic and the corresponding centrality.

Rank	Institution	Number	Centrality
1	Harvard University	53	0.19
2	University of Toronto	36	0.1
3	Brigham & Women’s Hospital	28	0.07
4	Institut National de la Sante et de la Recherche Medicale (Inserm)	23	0.1
5	Krembil Research Institute	22	0.11
6	University of London	20	0.12
7	Columbia University	15	0.03
8	University of California	14	0.07
9	Centre National de la	14	0.03
10	Massachusetts General	13	0.07

**Figure 5:**
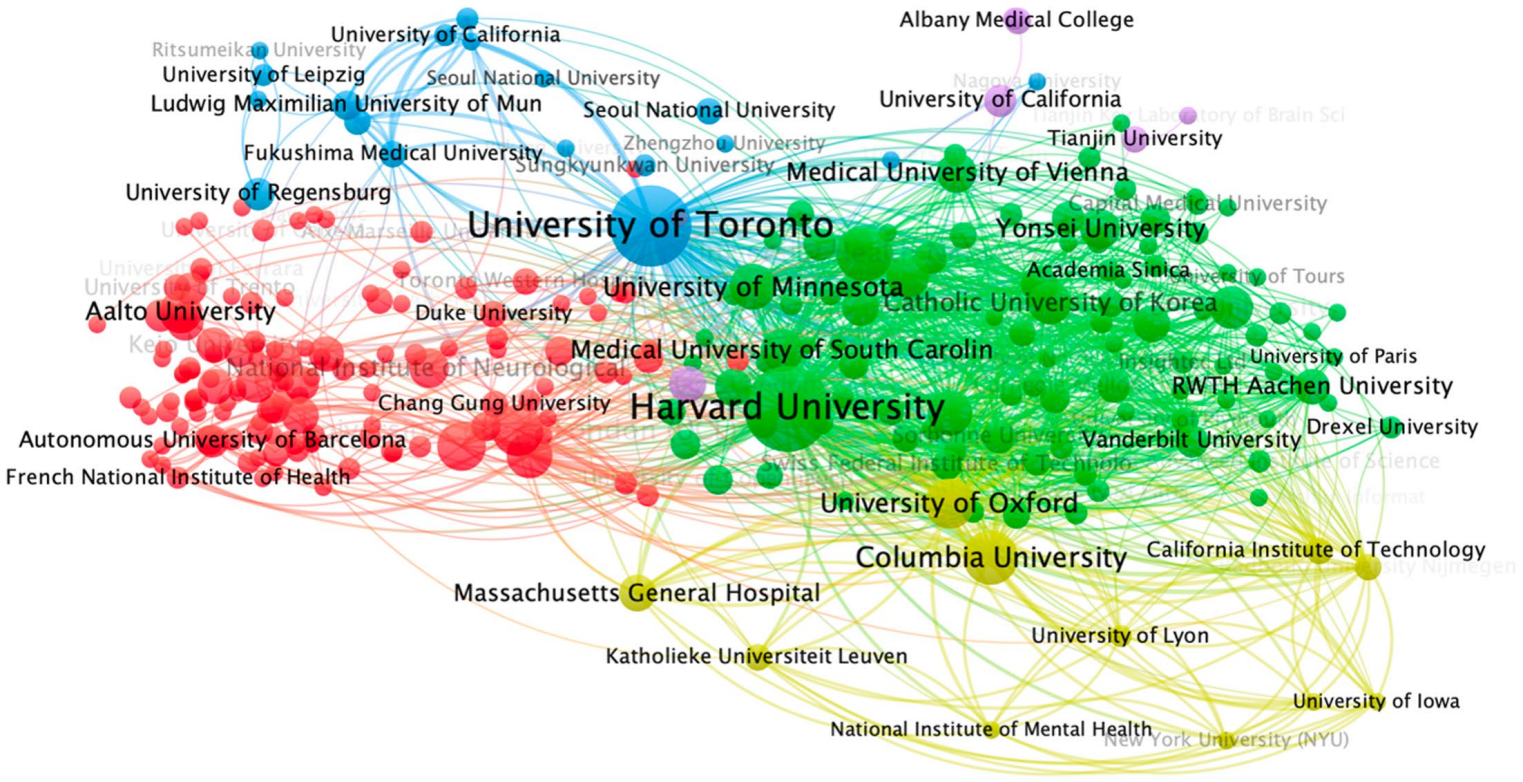
Network visualization of institutions. In the diagram, a circle represents an item, and each item corresponds to a label. The more important an item is, the larger its label and circle will be. Different colors indicate different clusters.

**Figure 6:**
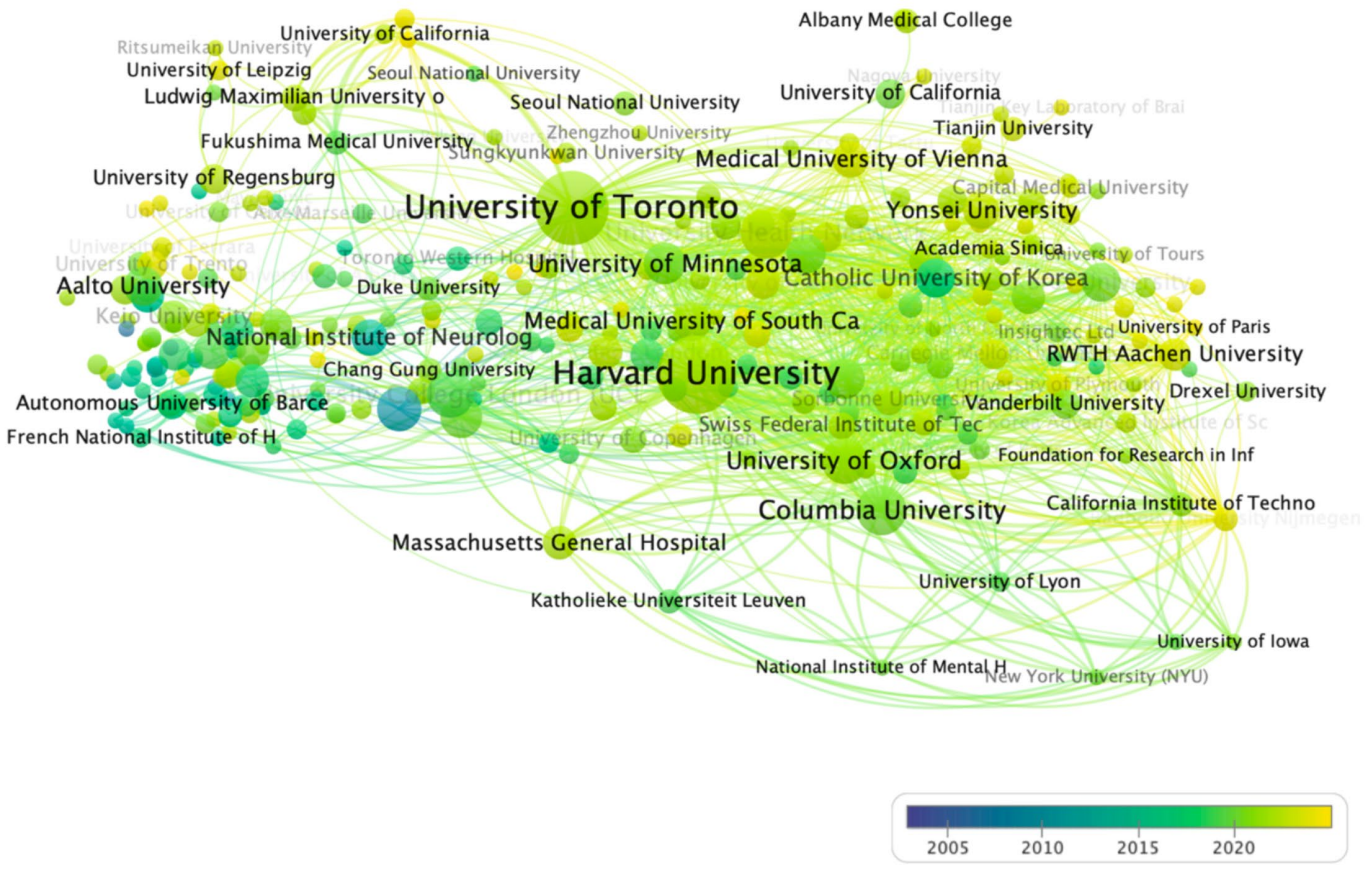
Overlay visualization of institutions (2004–2024). In the diagram, collaboration network of top 30 institutions, node size reflecting publication volume, edge thickness indicating collaboration strength, and color gradients representing temporal activity (yellow = recent, purple = earlier).

Figures 5, 6 depict the evolution of publication output across different institutions in the field of TUS from 2004 to 2024. Nodes colored in yellow indicate institutions with a higher volume of recent publications, reflecting more contemporary academic influence, while nodes in purple represent institutions with a greater number of earlier publications (de Castilhos et al., 2020). The figure highlights several academic institutions that have recently gained significant influence, including Harvard University, University of Toronto, and Anchen University. These institutions demonstrate a notable increase in research output and impact in recent years, underscoring their growing prominence in the TUS research landscape.

### 3.5 Authors cooperation network map

Table 3 presents the top 10 authors ranked by their publication output, affiliated institutions, and h-index. The author with the most publications is Chen Robert from University of Toronto, with 14 articles published. The author with the highest H-index is Lozano Andres M also from University of Toronto, with 144 H-index. This ranking highlights the most influential researchers in the field of TUS, as measured by their scholarly productivity and citation impact. The h-index, which reflects both the number of publications and their citation frequency, serves as a key metric for assessing academic influence. The table underscores the contributions of these leading authors and their respective institutions to advancing TUS research.

**Table 3:** The top 10 authors according to publications, the corresponding centrality, and H-index.

Rank	Author	Number	Institution	H-index	Subfields
1	Chen, Robert	14	University of Toronto	103	Neurosciences & Neurology
2	Yoo, Seung-Schik	13	Harvard University	55	Neurosciences & Neurology
3	Konofagou, Elisa E	11	Columbia University	77	Engineering
4	Lozano, Andres M	10	University of Toronto	144	Neurosciences & Neurology
5	Darmani, Ghazaleh	8	University Health Network	16	Neurosciences & Neurology
6	Aubry, Jean-Francois	8	ESPCI Paris	45	Radiology, Nuclear Medicine & Medical Imaging
7	Lee, Wonhye	7	Harvard University	27	Engineering
8	Kim, Hyungmin	6	Kyung Hee University	72	Engineering
9	Verhagen, Lennart	6	Radboud University Nijmegen	35	Neurosciences & Neurology
10	Sarica, Can	5	University of Toronto	15	Neurosciences & Neurology

### 3.6 Analysis of journal

#### 3.6.1 Journal and co-cited journals analysis

An analysis of journal and co-cited journals reveals the contributions to the field of TUS. Articles related to TUS have been published in 221 distinct journals. The top 10 journals, along with most co-cited journals, are in the Q1 quartile of JCR, characterized by high impact factors. This indicates that the quality of articles in the TUS field is exceptionally high. The journal with the highest number of publications and co-citations is *Brain Stimulation*, followed by *Clinical Neurophysiology* and *Neuroimage*. These findings underscore the significant influence and academic rigor of these leading journals in advancing TUS research (Table 4).

**Table 4:** The top 10 journal and co-cited journals.

Types	Rank	Name	Counts	IF 2023	JCR
Journal	1	*Brain Stimulation*	425	7.6	Q1
	2	*Clinical Neurophysiology*	350	3.7	Q1
	3	*Neuroimage*	343	4.7	Q1
	4	*Journal of Neuroscience*	321	4.4	Q1
	5	*Neuron*	321	14.7	Q1
	6	*Plos One*	314	2.9	Q1
	7	*Scientific Reports*	279	3.8	Q1
	8	*Nat Neuroscience*	235	21.3	Q1
	9	*Journal of Physiology-London*	227	4.7	Q1
	10	*Proceedings of the National Academy of Sciences of the United States of America*	226	9.6	Q1
Co-cited journals	1	*Brain Stimulation*	1,951	7.6	Q1
	2	*Clinical Neurophysiology*	1,333	3.7	Q1
	3	*Neuroimage*	1,057	4.7	Q1
	4	*Journal of Neuroscience*	875	4.4	Q1
	5	*Scientific Reports*	750	3.8	Q1
	6	*Neuron*	737	14.7	Q1
	7	*Journal of Physiology-London*	675	4.7	Q1
	8	*Ultrasound Medical Biology*	653	2.9	Q3
	9	*Neurology*	635	9.9	Q1
	10	*Experimental Brain Research*	606	1.9	Q4

#### 3.6.2 Dual-Map Overlay of journals

**Figure 7:**
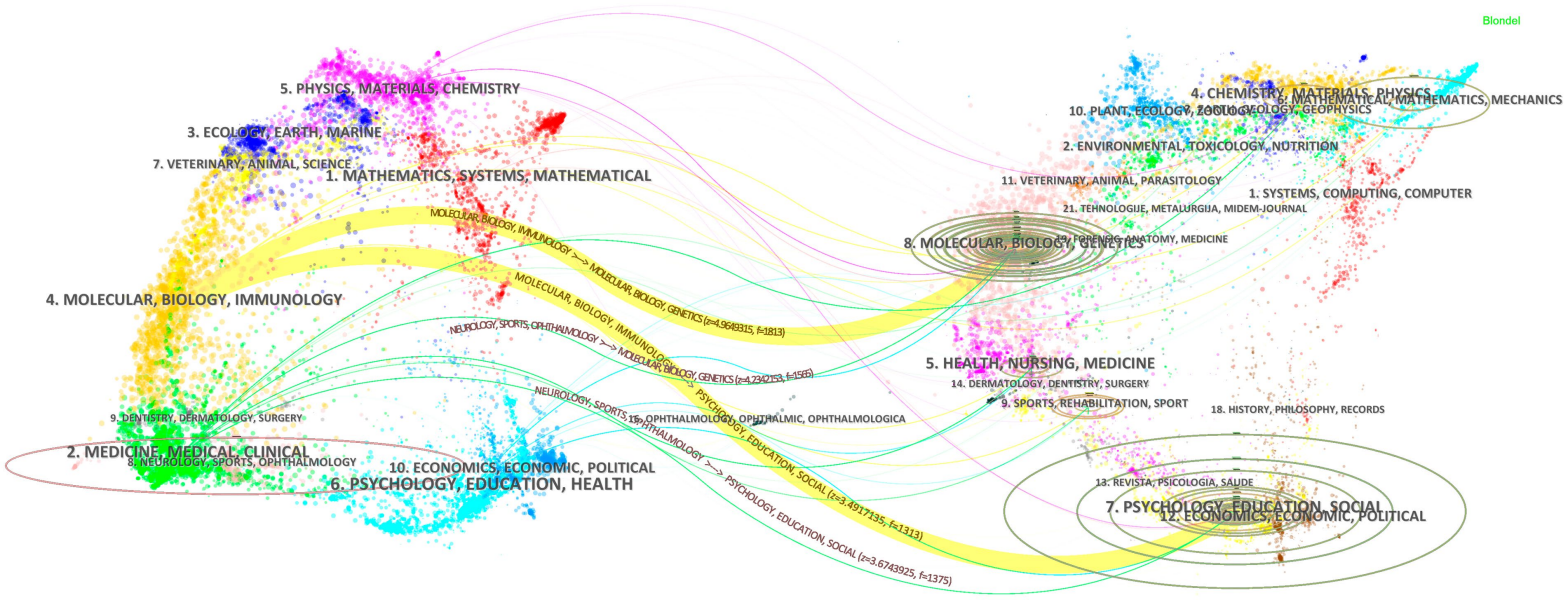
Dual-map overlay of article citations. The left chart shows the distribution of cited journals, classified by subject areas. The right figure depicts the distribution of cited journals. Similarly, they are classified by discipline. Wavy curves depict the citation links. Their colors are determined by their source star clusters. The communication and connection among the groups of cited journals indicate.

The Dual-Map Overlay visualizes citation relationships between distinct disciplinary domains, aiding researchers in understanding knowledge flow and cross-disciplinary influences (Chen and Leydesdorff, 2014). This analytical tool provides novel insights into the academic impact and intellectual contributions of research entities. In Figure 7, prominent yellow lines dominate as core citation pathways. The first yellow pathway links publications in journals categorized under MOLECULAR/BIOLOGY/IMMUNOLOGY to cited works in related domains. The green pathway connects journals in MEDICINE/MEDICAL/CLINICAL themes to their cited counterparts. These dominant pathways underscore the bidirectional knowledge exchange between foundational biological research (e.g., molecular mechanisms, immunology) and applied clinical studies (e.g., medical interventions, disease management).

### 3.7 Analysis of reference

#### 3.7.1 Reference co-occurrence analysis

Table 5 presents information on the top 10 highly cited articles in the field. The most frequent reference is Folloni et al. (2019), followed by Legon et al. (2018) and Verhagen et al. (2019). Among these, three studies were published in *Scientific Reports*. The most recent highly cited article is Beisteiner et al. (2020), which introduces an innovative therapeutic approach for neurodegenerative disorders.

**Table 5:** The top 10 highly cited reference.

Rank	Time cited	Reference (year)	Title	Journal (JCR)
1	53	Folloni et al. (2019)	*Manipulation of Subcortical and Deep Cortical Activity in the Primate Brain Using Transcranial Focused Ultrasound Stimulation*	*Neuron* (Q1)
2	49	Legon et al. (2018)	*Neuromodulation with single-element transcranial focused ultrasound in human thalamus*	*Human Brain Mapping*
3	49	Verhagen et al. (2019)	*Offline impact of transcranial focused ultrasound on cortical activation in primates*	*Elife*
4	48	Legon et al. (2018)	*Transcranial focused ultrasound neuromodulation of the human primary motor cortex*	*Scientific Reports* (Q1)
5	47	Blackmore et al. (2019)	*Ultrasound neuromodulation: a review of results, mechanisms, and safety*	*Ultrasound Medical Biology* (Q3)
6	41	Lee et al. (2016)	*Transcranial focused ultrasound stimulation of human primary visual cortex*	*Scientific Reports* (Q1)
7	40	Legon et al. (2014)	*Transcranial focused ultrasound modulates the activity of primary somatosensory cortex in humans*	*Nature Neuroscience* (Q1)
8	39	Sato et al. (2018)	*Ultrasonic Neuromodulation Causes Widespread Cortical Activation via an Indirect Auditory Mechanism*	*Neuron* (Q1)
9	39	Lee et al. (2016)	*Image-Guided Transcranial Focused Ultrasound Stimulates Human Primary Somatosensory Cortex*	*Scientific Reports* (Q1)
10	36	Beisteiner et al. (2020)	*Transcranial Pulse Stimulation with Ultrasound in Alzheimer’s Disease—A New Navigated Focal Brain Therapy*	*Advanced Science*

#### 3.7.2 Reference citation burst analysis

**Figure 8:**
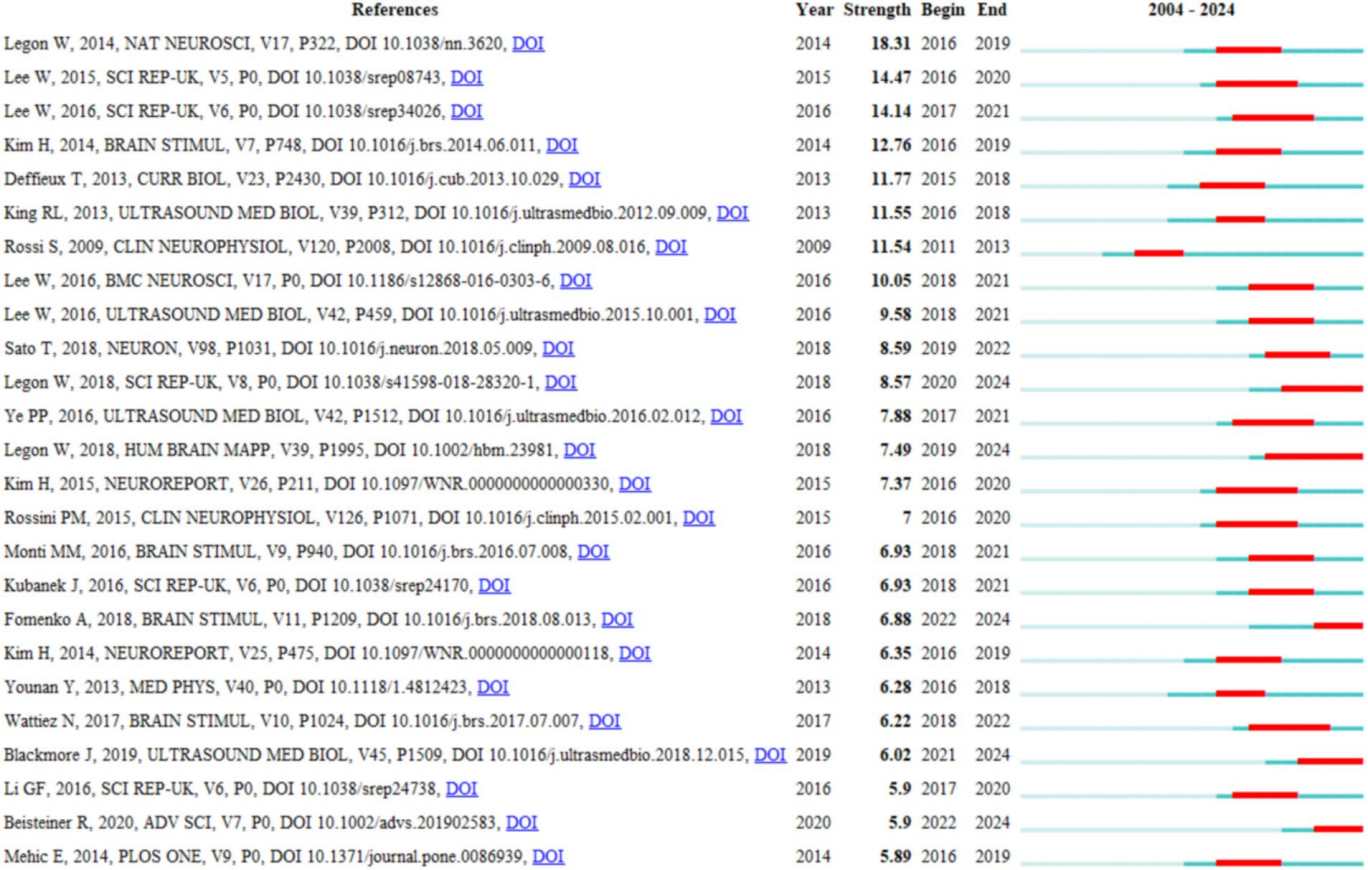
Top 25 references with the strongest citation bursts.

Reference bursts identify research hotspots and trends in the TUS field over defined time periods. Figure 8 displays the top 25 references with the strongest citation bursts. The blue bar spans the entire study period (2004~2024), while the red segment marks the duration of a reference’s burst. In the burst detection analysis: Start and End indicate the initiation and conclusion years of the burst. Strength quantifies the intensity of the burst, reflecting its statistical significance over time. The strongest citation burst was observed for the seminal work by Legon et al. (2014), published in *Nature Neuroscience* in 2014. This study, titled “*Transcranial focused ultrasound modulates the activity of primary somatosensory cortex in humans*,” demonstrated the potential of transcranial ultrasound for non-invasive neuromodulation, catalyzing widespread interest in TUS applications. Its sustained citation burst underscores its foundational role in shaping the field’s trajectory and inspiring subsequent mechanistic and clinical investigations.

### 3.8 Keywords analysis

#### 3.8.1 Keywords co-occurrence analysis

Table 6 lists the top 10 keywords based on their frequency of occurrence. The most frequent keyword is “transcranial magnetic stimulation,” followed by “non-invasive brain stimulation “, “brain stimulation “, “focused ultrasound “, and “cortex “.

**Table 6:** The top 10 keywords in terms of frequency.

Rank	Keywords	Frequency
1	Transcranial magnetic stimulation	202
2	Non-invasive brain stimulation	106
3	Brain stimulation	101
4	Focused ultrasound	81
5	Cortex	72
6	Excitation	61
7	Deep brain stimulation	57
8	Motor cortex	51
9	Repeat transcranial magnetic stimulation	50
10	Brain	49

**Figure 9:**
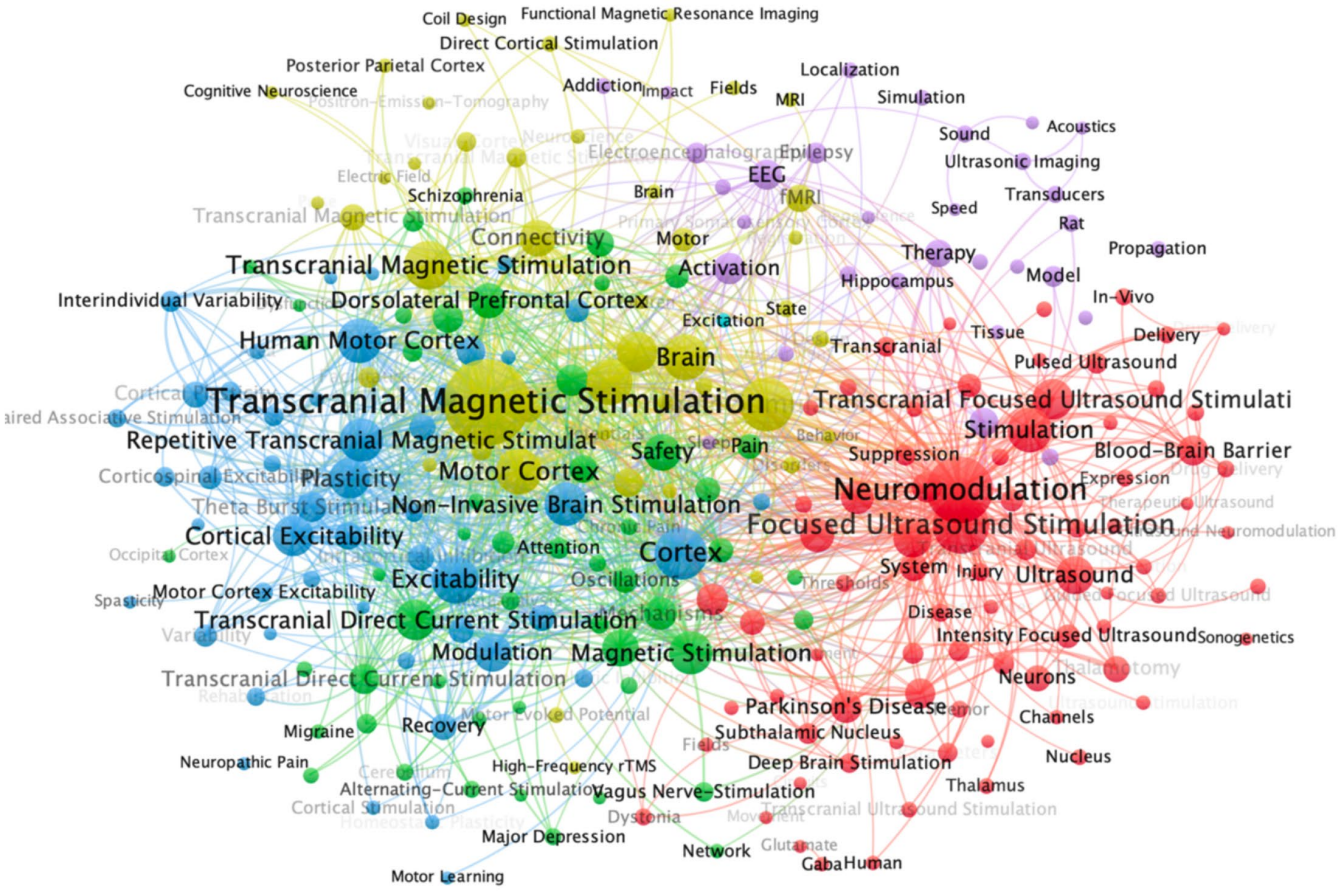
Co-occurrence network visualization of keywords. In the diagram, a circle represents an item, and each item corresponds to a label. The more important an item is, the larger its label and circle will be. Figure presents a network visualization of keywords, where nodes of different colors represent keywords clustered into distinct thematic groups, and node size corresponds to their frequency of occurrence.

**Figure 10:**
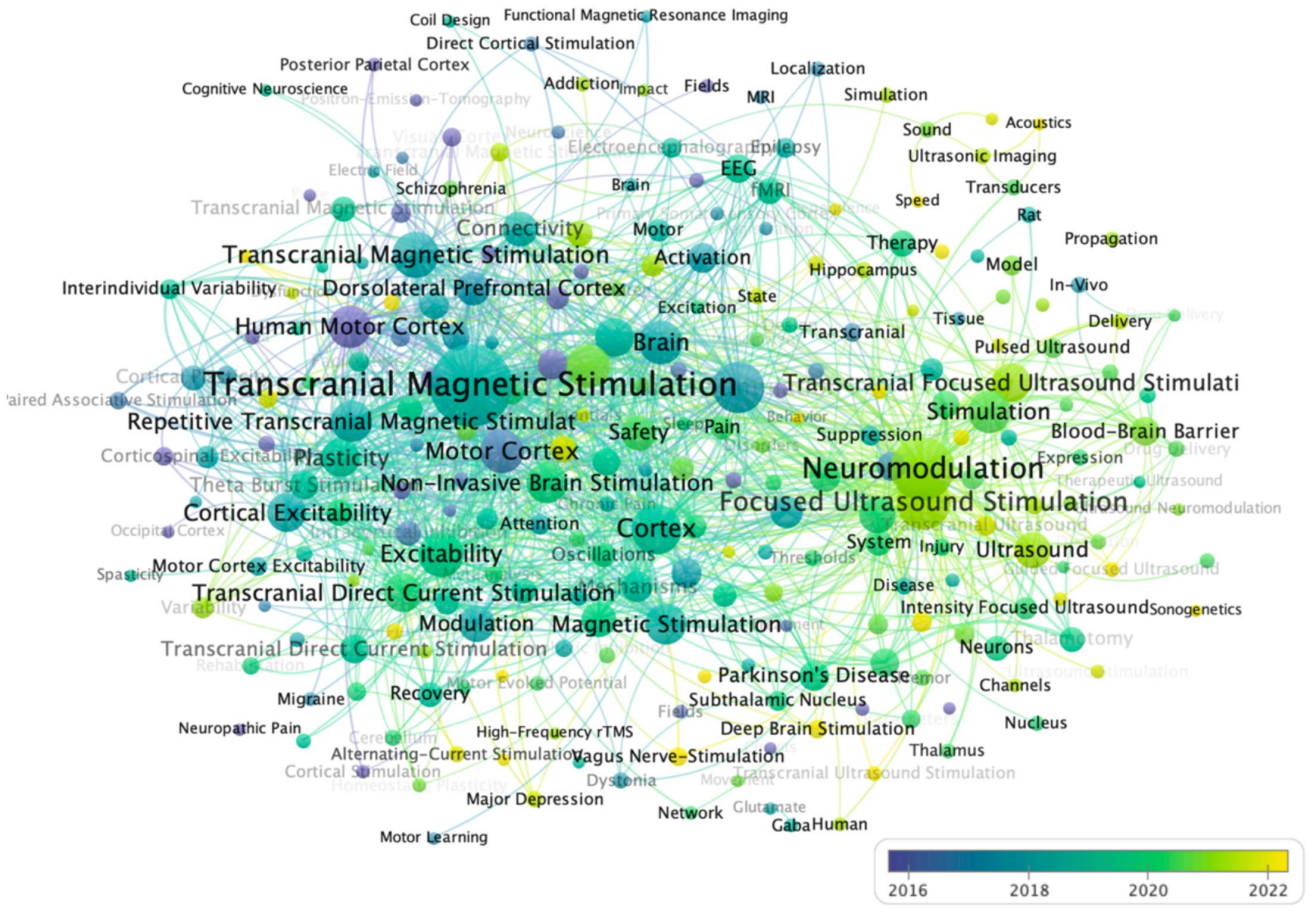
Co-occurrence overlay visualization of keywords in VOSviewer. Figure displays an overlay visualization of keywords, with nodes colored in yellow denoting recently emerging keywords, reflecting contemporary research trends, while purple nodes represent earlier keywords, highlighting foundational themes.

**Figure 11:**
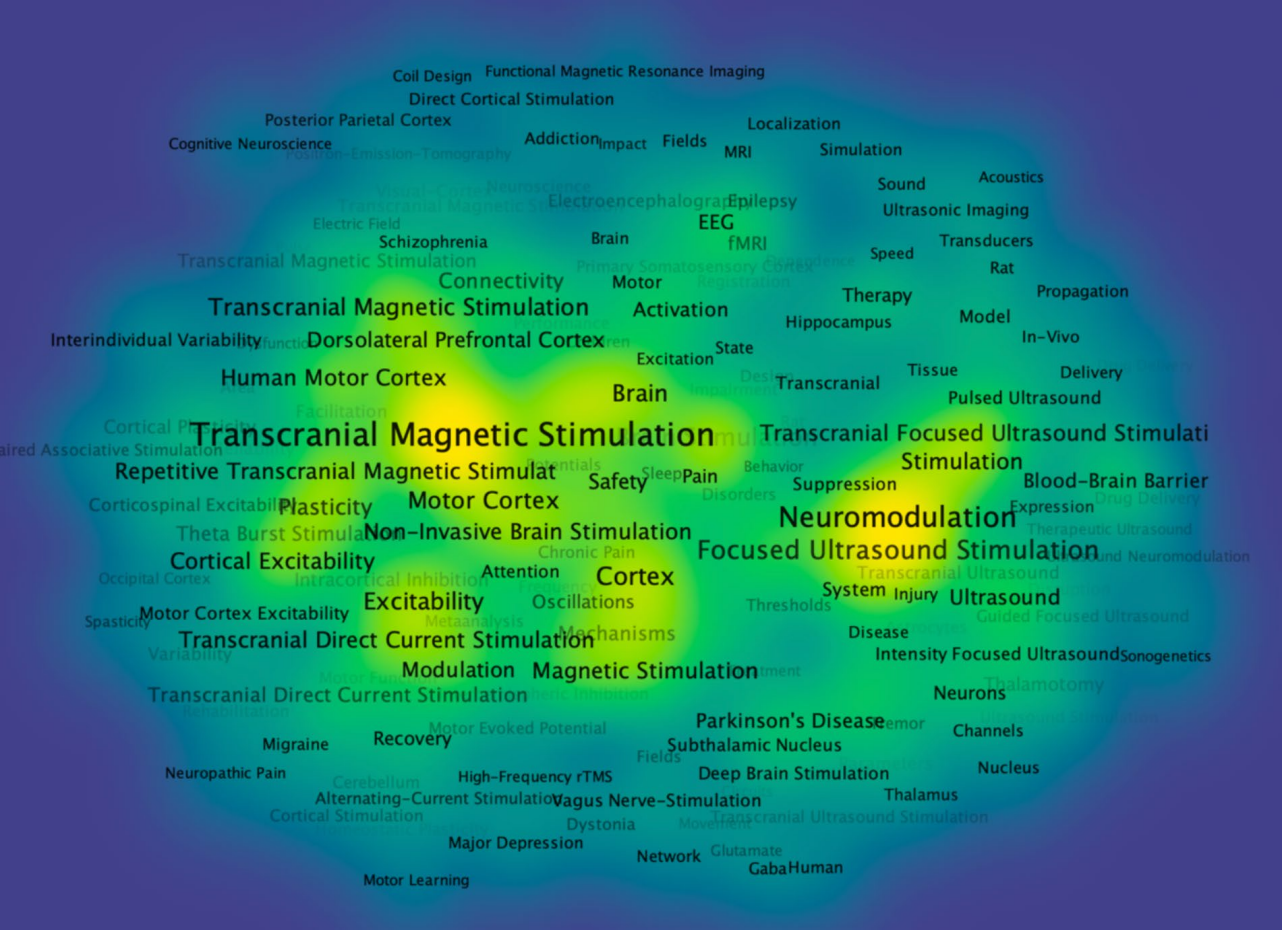
Co-occurrence density visualization of keywords in VOSviewer. Figure provides a density visualization of keywords in the form of a heatmap, where warmer colors indicate regions of higher keyword density, illustrating areas of concentrated research activity.

Figures 9–11 present a comprehensive bibliometric visualization of keyword networks and their temporal evolution in transcranial stimulation research. Figure 9 displays the keyword co-occurrence network, revealing two dominant clusters centered around “transcranial magnetic stimulation “and “neuromodulation.” These core nodes are surrounded by related terms including “focused ultrasound stimulation,” “brain,” “cortex,” “activation,” and “transcranial direct current stimulation,” forming a cohesive thematic structure that reflects current research directions in the field. The temporal analysis in Figure 10 demonstrates a clear shift in research focus over time. Early studies predominantly concentrated on “transcranial magnetic stimulation,” while recent investigations have progressively emphasized “neuromodulation” and “focused ultrasound stimulation,” indicating an evolving research paradigm in non-invasive brain stimulation techniques. Complementing these findings, Figure 11 presents a density visualization of keyword distribution. The heatmap highlights “transcranial magnetic stimulation” and “neuromodulation” as the most intensive research areas, confirming their central position in this scientific domain.

#### 3.8.2 Keywords clusters analysis

**Figure 12:**
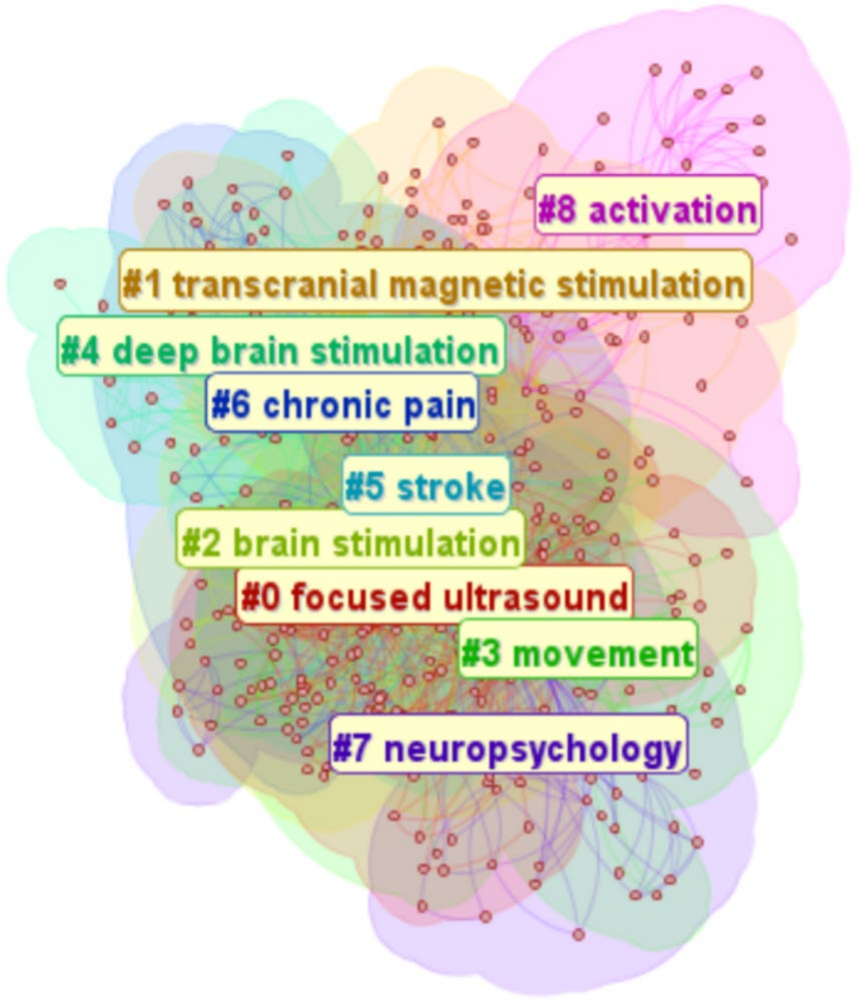
High-frequency keywords cluster map. The figure shows the keyword clustering analysis of the included Chinese literature. Each color represents a cluster, and the size of the category affects the ranking.

The keyword cluster map emphasizes the structural relationships between clusters, highlighting critical nodes and significant connections. Figure 12 presents a keyword cluster analysis of the included Chinese literature, identifying nine distinct clusters: #0 focused ultrasound, #1 transcranial magnetic stimulation, #2 brain stimulation, #3: movement, #4: deep brain stimulation, #5: stroke, #6: chronic pain, #7: neuropsychology, #8: activation. These metrics confirm the high credibility and structural rationality of the clustering results, with the Q value indicating significant modular structure and the S value reflecting strong homogeneity within clusters. The modularity (Q) and silhouette (S) scores for the keyword clusters were calculated as 0.429 (Q > 0.3) and 0.746 (S > 0.7), respectively.

#### 3.8.3 Keywords citation burst analysis

**Figure 13:**
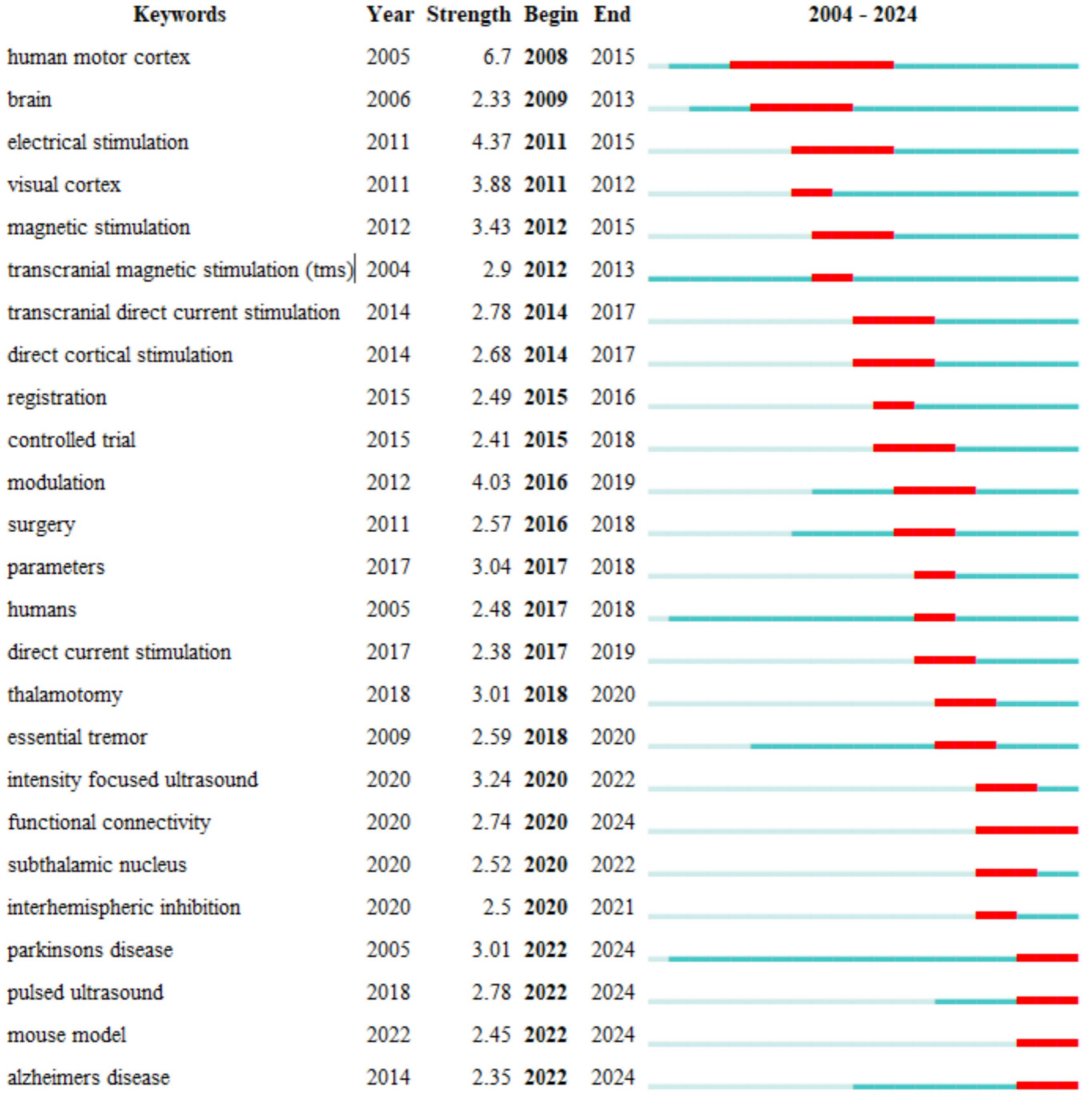
Top 25 keywords with the strongest citation burst.

Keyword bursts reflect research hotspots and trends within the TUS field over specific periods. Figure 13 displays the top 25 keywords with the strongest citation bursts. Among these “human motor cortex” exhibited the highest burst strength (6.7) over the past two decades. Keywords with bursts persisting into 2024 include “functional connectivity” “Parkinson’s disease” “pulsed ultrasound” “mouse models” and “Alzheimer’s disease” highlighting emerging research priorities and technological advancements in the field. These bursts underscore the dynamic evolution of TUS research particularly its growing focus on neurological disorders mechanistic studies and translational applications.

## 4 Discussion

This study employs bibliometric analysis to investigate the status and developmental trends of TUS research. By visualizing and analyzing data from articles published between 2004 and 2024, including country/region contributions, institutional and author collaborations, journal distributions, citation networks, and keyword co-occurrences, this work identifies global research hotspots and emerging trends. The findings of this study will assist researchers in pinpointing key areas of interest within the field and selecting appropriate research directions for future investigations.

### 4.1 Current research landscape in the global TUS field

#### 4.1.1 Growth trends in TUS research

TUS has emerged as a rapidly evolving field with significant potential for non-invasive neuromodulation and therapeutic applications. The bibliometric analysis of TUS research from 2004 to 2024 reveals a significant upward trend in publication volume, particularly after 2013. The quadratic growth model indicates an accelerating interest in TUS, with a peak in 2024. The number of TUS-related publications has shown a steady increase, with a notable acceleration in the past decade. This growth reflects the increasing recognition of TUS as a promising non-invasive neuromodulation technique, particularly in the fields of neuroscience and rehabilitation.

#### 4.1.2 Global cooperation, institutional and author influence in TUS research

In an analysis of global collaborations, contributions from the United States, China, and Germany were found to dominate, reflecting their strategic investments in neurotechnology and translational research. The collaborative network map further highlights the centrality of the United States, Canada, and South Korea in promoting international partnerships. Harvard University, the University of Toronto, and Brigham & Women’s Hospital emerged as leading institutions, with Harvard uniquely excelling in both high productivity and high network centrality. This dual dominance implies its role as a hub for both high-output research and knowledge dissemination. Author analysis revealed Chen Robert and Lozano Andres M as key contributors, emphasizing the University of Toronto’s leadership in translational TUS studies. Such institutional and individual prominence highlights the importance of specialized research centers in advancing methodological and clinical breakthroughs.

In this study, Interdisciplinary and cross-border collaboration is important for TUS research, with the main goal of integrating multidisciplinary resources to achieve more accurate, safe, and effective neuromodulation therapy. The collaboration involves a variety of disciplines including neuroscience, medicine, biophysics, and engineering. The strengths of each discipline complement each other, in the direction of theoretical basis, clinical needs and mechanism of action, to improve the focus accuracy and treatment effect of TUS. Research institutions have unique advantages in interdisciplinary and cross-border collaboration. For example, Harvard University has strong scientific research strength attracts the talent, has advanced experimental equipment, and maintains close cooperation with many international institutions, and plays an important leading role in the technological innovation and clinical application expansion of TUS research. In the future TUS research, we should actively promote the exchange and cooperation between researchers from different disciplines, build an interdisciplinary research platform, and promote the integration and innovation of knowledge and technology. At the same time, strengthen international cooperation, share resources, and research results, achieve complementary advantages, promote the rapid development of TUS research, so that it can be better applied to the clinic for the benefit of patients.

#### 4.1.3 Journal impact and citation status

Many articles in TUS field are published in high-impact journals such as *Brain Stimulation*, *Clinical Neurophysiology*, and *Neuroimage*. All three journals consistently rank in the Q1 of JCR, reflecting their high academic credibility and global recognition. *Brain Stimulation* publishes pervious research on non-invasive neuromodulation, including TUS, TMS, tDCS. The key topics include optimization of stimulation parameters, mechanistic studies on neural excitability and synaptic plasticity and clinical applications for neurological and psychiatric. Rising publication numbers in these journals reflect growing interest from both academic and clinical communities.

In the top 10 cited article, most involving TUS relation with brain function, including three articles published in the *Scientific Reports*. The most cited article is Folloni et al. (2019), in which the authors used fTUS to manipulate subcortical and deep cortical activity in primates, and the study found that TUS can affect the activities of deep brain regions. The most recent highly cited article is Beisteiner et al. (2020). This research underscores the growing interest in non-invasive neuromodulation techniques and their translational potential in addressing Alzheimer’s disease. The prominence of these studies reflects their significant impact on advancing both methodological innovations and clinical applications in TUS research.

### 4.2 Research hotspots

Based on the co-occurrence analysis of keywords, cluster analysis and citation burst analysis, the current research hotspots of TUS were divided into the following three aspects.

#### 4.2.1 Non-invasive neuromodulation

TUS is a novel non-invasive neuromodulation technique with promising efficacy and potential clinical applications. The optimization of TUS parameters (e.g., frequency, intensity, duty circle) has emerged as critical research hotspots, as evidence by the high-frequency keywords “focused ultrasound” (*n* = 81) and “excitation” (*n* = 61) in our analysis. Fundamental frequency is a key determinant of TUS efficacy (Lee et al., 2016; Fomenko et al., 2018), influencing both penetration depth and spatial resolution. Studies suggest that low-frequency TUS is generally considered more suitable for stimulating deep brain regions (Tufail et al., 2010; Menz et al., 2019), while high-frequency TUS is appropriate for spatial resolution (Pichardo et al., 2011). The citation burst analysis further highlights “human motor cortex” (strength = 6.71) as a sustained research focus. TUS was used to modulate brain regions activity and treating neurological disorders (Grippe et al., 2024; Osou et al., 2024). For instance, TUS applied to the hippocampus in Alzheimer’s disease patients improved memory, executive function, and cognitive ability without opening the blood–brain barrier, a finding strongly supported by the high citations of Beisteiner et al. (2020) (*n* = 36). In Parkinson’s disease studies, TUS has shown positive effects on motor cortical areas, and UPDRS-III scores showed a decrease in the score (Grippe et al., 2024; Osou et al., 2024). In particular, TPS has shown promise in balancing safety and efficacy by minimizing thermal effects while maintaining neuromodulator outcomes (Beisteiner et al., 2020).

#### 4.2.2 Therapeutic applications

The translational potential of TUS is increasingly evident in diverse clinical domains, reflected by the keyword clusters “#5 stroke” and “#6 chronic pain” in our analysis. In stroke rehabilitation, TUS targeting the motor cortex improved upper limb motor recovery by 25% compared to sham controls in a randomized study (Lee et al., 2016). This aligns with the dominance of American–China–Germany collaborations in driving multicenter trails. For chronic pain, used TUS to stimulate the PFC and ACC of patients with chronic neuropathic pain, relieving pain symptoms and improving patient mood (Hameroff et al., 2013; Shin et al., 2023). This topic highlighted by the citation burst of Hameroff et al. (2013). In the field of psychiatric and psychological disorders, preliminary evidence suggests that TUS modulation of the prefrontal cortex alleviates depressive symptoms by normalizing default mode network connectivity (Reznik et al., 2020; Oh et al., 2024; Schachtner et al., 2024), supported by emerging keywords “functional connectivity” and “Alzheimer’s disease.”

A groundbreaking frontier lies in TUS-mediated blood–brain barrier (BBB) modulation (Elias et al., 2016), which correlates with the recent surge in publications from institutions like Harvard University and the University of Toronto. Preclinical studies have shown that low-intensity pulsed ultrasound combined with microvesicles can instantly open the blood–brain barrier, and MRgFUS therapy targeting the ventromedial thalamic nucleus (VIM) can reduce tremor in patients with Parkinson’s disease, and the effect can last for at least 1 year (Bond et al., 2017). However, the bibliometric results reveal limited international collaboration on safety protocols, underscoring the need for rigorous evaluations to avoid off-target effects.

#### 4.2.3 Mechanistic studies

Elucidating the biophysical mechanisms of TUS is pivotal for refining its therapeutic applications. Mechano transduction via activation of Piezo1 ion channels has been identified as a key pathway for ultrasound-induced neuronal excitation (Zhu et al., 2023). Additionally, TUS enhances synaptic plasticity by upregulating BDNF expression in the hippocampus, as shown in murine models of cognitive impairment (Lin et al., 2015; Huang et al., 2017), a mechanistic focus reflected in the high centrality of journals like *Neuron*. Preclinical safety studies in rodents reveal that exposure to intensities below I_SPTA_ = 3 W/cm^2^ causes no histological damage, establishing preliminary safety thresholds (Wattiez et al., 2017), yet the bibliometric analysis shows minimal contributions from low/middle-income courtiers. However, interspecies differences in skull attenuation necessitate caution when extrapolating results. Future mechanistic work should prioritize human-derived neural models and real-time neuroimaging to map dose–response relationships.

#### 4.2.4 Safety and off-target effects

Studies have shown that TUS has significant therapeutic potential, and a rigorous safety assessment is urgently needed. Due to the significant differences in individual skull structure and acoustic characteristics, it is difficult to ensure the establishment of a uniform safety standard for TUS. At the same time, due to the lack of long-term, large-scale clinical study data, it is challenging to accurately evaluate the long-term safety of TUS.

Some studies have reported adverse reactions, including headache (Beisteiner et al., 2020; Cheung et al., 2023a; Hameroff et al., 2013; Osou et al., 2024; Cheung et al., 2023b; Cheung et al., 2024), fatigue (Osou et al., 2024; Samuel et al., 2023), nausea and vomiting (Cont et al., 2022; Cheung et al., 2023a), naming and memory impairment (Lee et al., 2022), swelling and scalp fever (Lee et al., 2022; Mahoney et al., 2023), worsening mood (Beisteiner et al., 2020), drowsiness and pain (Cont et al., 2022). However, all these adverse reactions were transient nature and mild in severity. In some animal studies, abnormal visual responses have been observed after TUS exposure (Wattiez et al., 2017). To minimize the effect of miss distance, the first design should focus on precision of the ultrasonic equipment, ensure energy precisely to the target area (Dallapiazza et al., 2018). Second, the stimulation parameters (including frequency, intensity, and duration time) are optimized to achieve a therapeutic effect (Legon et al., 2014; Tyler et al., 2018). In addition, the use of imaging guidance techniques, such as magnetic resonance imaging (MRI) guidance, can help to monitor TUS treatment in real time and thus adjust the treatment plan in time (Elias et al., 2016; Gallay et al., 2021). Therefore, international cooperation on security protocols is essential to overcome these barriers and promote safe and effective clinical use of TUS.

### 4.3 Emerging trends and future directions

#### 4.3.1 Technological advancements

The integration of TUS with other NIBS technologies represents a significant advancement in the field (Darmani et al., 2022). This includes the development of hybrid systems that combine TUS with functional magnetic resonance imaging (fMRI) (Ai et al., 2018) or electroencephalography (EEG) (Legon et al., 2018; Lee et al., 2016; Mueller et al., 2014), enabling real-time monitoring of neuromodulator effects. Additionally, the synergy between TUS and established techniques such as transcranial magnetic stimulation (TMS) is being explored to enhance therapeutic precision and efficacy (Thielscher and Kammer, 2004; Huang et al., 2011). In addition, the combination of artificial intelligence and patient-specific targeting is expected to improve spatial resolution and temporal accuracy, enabling personalized treatment approaches. In terms of spatial resolution, AI can analyze large amounts of data from imaging methods such as MRI and CT and is able to distinguish various brain tissues with high precision, which helps to precisely direct ultrasound waves to predetermined area (Kulathilake et al., 2025). In terms of temporal accuracy, AI can leverage a patient’s real-time physiological data, such as electroencephalogram (EEG) signals and blood flow changes measured by functional near infrared spectroscopy (fNIR), which can be analyzed in real time to predict the optimal timing of TUS stimulation, triggering TUS treatment at the right moment, thereby maximizing treatment effectiveness (Luo et al., 2024). In addition, the AI can also customize the time pattern of stimulation pulses. According to the unique characteristics of different diseases and patients, personalized stimulus sequences are designed to improve the therapeutic effect.

#### 4.3.2 Clinical translational gaps

Most of the previous studies have been small sample studies, so expanded clinical trials are needed to validate the efficacy of TUS in different populations, including age-specific cohort studies such as older patients and patients with comorbidities (Sarica et al., 2022). This will ensure that TUS therapies are both effective and broadly applicable. At the same time, the current research conclusions are mainly from animal models, and human neurophysiology needs to be verified in the future (Darmani et al., 2022).

#### 4.3.3 Parameter standardization

Currently, optimal TUS parameters vary significantly across different applications, which hinders clinical adoption (Nandi et al., 2024). To address this, multicenter trials are essential to identify disease-specific parameter thresholds. Additionally, the development of consensus guidelines through international collaborations with organizations such as the World Health Organization (WHO) and the Institute of Electrical and Electronics Engineers (IEEE) is crucial. These efforts will standardize protocols, ensuring reproducibility and safety across various clinical settings.

#### 4.3.4 Long-term safety and efficacy

While existing evidence primarily focuses on short-term outcomes, longitudinal studies are critical to assess the risks associated with chronic exposure, such as microhemorrhage, tissue heating, or unintended neuroplastic changes. Establishing registries to monitor adverse events and the durability of therapeutic benefits over extended periods is imperative (Pellow et al., 2024). Collaboration with regulatory bodies, including the Food and Drug Administration (FDA) and the European Medicines Agency (EMA), will be essential to establish approval pathways for TUS-based therapies, ensuring their safe and effective integration into clinical practice.

## 5 Limitations

This study provides a comprehensive bibliometric overview, several limitations must be acknowledged: ① Database bias: Reliance on WoSCC may exclude non-indexed or regional journals; ② Temporal Constraints: A time limit of nearly two decades may ignore the effects of early pioneering studies; ③ The omission of non-English articles: This analysis was limited to English-language publications indexed in WoS, which may underrepresent research published in other languages.

## 6 Conclusion

Transcranial ultrasound stimulation, as a non-invasive neuromodulation technique, has attracted increasing attention from researchers and has become increasingly prominent in neuroscience research. At present, the application of TUS mainly focuses on neurological diseases, among which the research on Parkinson’s disease and Alzheimer’s disease is the most extensive. This study finds that in terms of international cooperation, a promotion for the development of TUS research has been formed with the United States, China and Germany as the core. At present, although preclinical studies have demonstrated promising neuroregulatory effects, translating these findings into clinical practice requires further verification through multi-center trials to ensure effectiveness, safety and repeatability. This study provides ideas for future research and promotes the development of TUS.

## Data Availability

The datasets presented in this study can be found in online repositories. The names of the repository/repositories and accession number(s) can be found in the article/supplementary material.
